# Correction

**DOI:** 10.1080/13880209.2024.2323905

**Published:** 2024-02-29

**Article title:** Physcion inhibition of CYP2C9, 2D6 and 3A4 in human liver microsomes

**Authors:** Liu, L., Sun, S., & Li, X.

**Journal:** *Pharmaceutical Biology*

**Bibliometrics:** Volume 62, Number 1, pages 207–213

**DOI:** https://doi.org/10.1080/13880209.2024.2314089

The above-mentioned paper contains the below errors when it was first published online.

1. The legend inside Figure 5A was incorrect. This has now been corrected and Figure 5 has been reproduced below.

**Figure:**
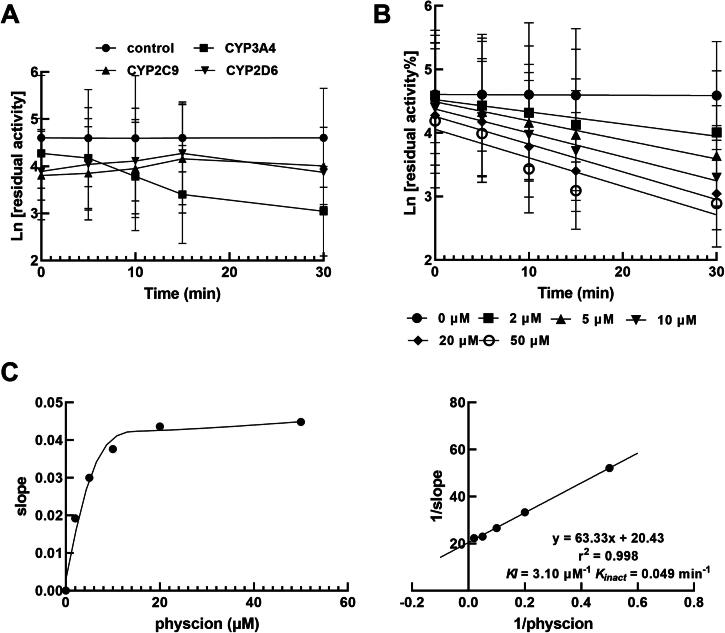
2. The following sentence “The activity of CYP450 isoforms was evaluated by a cocktail assay with…Carrão et al. 2018).” was incorrect in the sub-section “Study design”. This has now been corrected as “The activity of CYP450 isoforms was evaluated with…Carrão et al. 2018).” where the text “by a cocktail assay” has been removed.
3. The following sentence "This study employed a cocktail assay, which is an indirect method." was incorrectly included in the 4th paragraph under the "Discussion" section which has now been removed.

The above errors have been corrected and the article has been republished online.

